# Methods of Passenger Ride Comfort Evaluation—Tests for Metro Cars

**DOI:** 10.3390/s23125741

**Published:** 2023-06-20

**Authors:** Róża Wawryszczuk, Ewa Kardas-Cinal, Jerzy Lejk, Marek Sokołowski

**Affiliations:** 1 Faculty of Transport, Warsaw University of Technology, 00-662 Warsaw, Poland; ewa.kardascinal@pw.edu.pl; 2 Faculty of Civil Engineering, Warsaw University of Technology, 00-637 Warsaw, Poland; jerzy.lejk@pw.edu.pl; 3 Warsaw Metro, 02-798 Warszawa, Poland; m.sokolowski@metro.waw.pl

**Keywords:** ride comfort, metro car, public transport, vibrations

## Abstract

Ride comfort is one of the key issues in passenger transport. Its level depends on many factors related to both environmental factors and individual human characteristics. Ensuring good travel conditions translates into higher quality transport services. This article presents a literature review, which shows that ride comfort is most often considered in the context of the impact of mechanical vibrations on the human body, while other factors are usually neglected. The aim of this study was to conduct experimental studies that take into account more than one type of ride comfort. These studies concerned metro cars in the Warsaw metro system. Three types of comfort were evaluated: vibrational, thermal, and visual, based on vibration acceleration measurements, air temperature, relative air humidity, and illuminance. The ride comfort in the vehicle bodies’ front, middle, and rear parts was tested under typical running conditions. The criteria for assessing the effect of individual physical factors on ride comfort were selected based on applicable European and international standards. The test results indicate good thermal and light environment conditions in every measuring point. The slight decrease in passenger comfort is undoubtedly due to the effects of vibrations occurring while mid journey. In tested metro cars, horizontal components have a more significant impact on reducing vibration comfort than other components.

## 1. Introduction

Ride comfort is a complex concept and depends on many factors. The level of ride comfort depends on factors characterizing the passenger’s environment, such as vibrations, noise, air temperature and relative humidity, lighting, and pressure. Ergonomics of the seat or spatial layout of the vehicle interior and the time the passenger is exposed to the impact of individual environmental factors are also important. It should be mentioned that the feeling of comfort is subjective and may vary depending on a person’s age, weight, height, and other characteristic features. Due to differences in the individual perception of different stimuli occurring during the journey, an assessment of passenger ride comfort is not straightforward.

Statistics on passenger transport by rail show consumers’ growing interest in these services. According to the Polish Office of Rail Transport statement, in January 2022, nearly 22 million people traveled by rail in Poland. This number increased by 38% in the same period of 2023 [1]. Despite a clear upward trend in rail passenger transport, many people still choose the car as a means of transportation for everyday and long-distance journeys. Changing habits within society is necessary mainly for environmental reasons, but also for better urban planning and limiting the negative impact of transport on public health [2]. The change requires many actions, which should primarily be aimed at improving the quality of public transport services. The EN 13816:2002 standard [3] lists the criteria for assessing the quality of these services in relation to various factors, including spatiotemporal availability, safety, and comfort. Given the above, the issue of ride comfort remains a current and vital topic.

The aim of this article is to identify the dominant factors affecting the level of passenger comfort for rail transport and their assessment based on the recommendations of currently applicable standards. This article reviews the literature in the comfort field and presents the results of experimental research carried out in selected types of metro cars operating within the Warsaw metro system.

Assessment of ride comfort in rail vehicles is often an additional part of running safety tests. Therefore, the concept of ride comfort is most often identified with vibrational comfort. To the best knowledge of the authors there are only a few studies that assess other types of comfort, and even less that take into account more than one stimuli. This article adds a broader view on the issue of ride comfort in the Warsaw metro. It shows good visual, thermal, and vibrational conditions with slightly reduced comfort related to vibrations in the center part of the tested metro car. The use of applicable standards allows for the comparison of comfort levels in various metro systems around the world.

## 2. Literature Review

Scientific research in the field of ride comfort is most often focused on the assessment of one factor affecting the passenger experience. This factor is the mechanical vibrations of the vehicle body influencing the passenger during the vehicle’s movement. Among the many studies on the topic, researchers use various methods and approaches, where the effects of vibrations on the passenger are assessed based on measured vibration acceleration courses or obtained as a result of simulation. The ride comfort is also considered when investigating other phenomena such as the running safety and dynamic responses of a vehicle body [4].

An example is an article in which the authors propose a research methodology using simulation models that consider the realistic traffic congestion conditions of various types of road vehicles moving on bridges with a large span [5]. To obtain more accurate dynamic responses, in addition to the vibrations caused by the interaction between the bridge and the vehicles, the effects resulting from the occurrence of crosswinds have also been taken into account. To evaluate the impact of the vibration acceleration obtained in such simulations on ride comfort, the weighted method from the ISO 2631-1 standard [6] was used. Comfort indicators for cars and trucks were determined based on vibrations occurring at three contact points between the human body and the vehicle: floor, seat, and backrest. The same standard was used in study [7] but for assessing vibrational comfort in urban, suburban, and long-distance rail vehicles.

The weighted method was also used in study [8]. The authors aimed to identify zones with reduced vibrational comfort in a long-distance bus. The study used a vehicle simulation model, for which the values of comfort indicators on the seats for passengers and drivers were determined, taking into account the different speeds of vehicles moving on roads with asphalt concrete surfaces in good and bad condition. In addition, the authors analyzed the impact of changes in seat parameters (stiffness and damping) on the improvement of passenger comfort in individual zones.

Another example of simulation studies can also be found in study [9], where the comfort associated with the impact of vibrations induced in cars during passage through a railway junction was assessed. For the analysis, a “quarter car” model was used, which was calibrated with the help of vibration measurements on real road sections with speed control bumps of known profiles and different sizes. A comprehensive simulation model describing the dynamics of high-speed rail vehicles is presented in the article [10]. As in the previously cited study, the authors developed a vehicle model, this time including a train-seat-human system. Then, they compared the ride comfort resulting from vibrations present on the floor and the seat of the vehicle in different places on the train. Only vertical vibrations were considered in the analysis, assuming that these vibrations are the most important in the context of the vibrational comfort of the passenger.

Similar studies aimed at specifying which seats in the vehicle contribute to vibration discomfort are presented in article [11]. This time the object of study was three trams of the same type but with different mileage. The European standard EN 12299:2009 [12] was used to assess ride comfort. The authors of the study presented the results of experimental research carried out on the reference track based on measurements recorded on the vehicle’s floor and selected seats. The recommendations of the above-mentioned European standard were also used in study [13] presenting the results of ride comfort in a model train depending on stiffness and damping parameters of the primary and secondary suspension systems.

A different approach to vibrational comfort is presented in article [14] where the authors investigated the effect of idle vibrations which are felt by passengers when a road vehicle stops with the engine running and are due to the engine vibrations. The proposed methodology was based on measurements in the test vehicle, which were then reconstructed at a vibrating station equipped with the same seat as the reference car. Nine subjects were put to vibration and asked to rate the level of discomfort when exposed to sinusoidal test signals and signals corresponding to idle vibrations. The vibration excitation station was also used in study [15], which concerned the subjective assessment of vibration comfort in three selected road vehicles. The experiment involved a larger group of people and considered the effects of vibrations not only on the seat but also on the floor and steering wheel.

Researchers are also trying to develop existing methods of assessing comfort, as it is presented in article [16], where a new CI index (comfort index) taking into account the speed and type of road vehicle is proposed and is based on the index from ISO 2631-1:1997 standard [6]. The obtained result, as in the cited standard, is compared with a six-point comfort scale, but the thresholds of individual levels have changed. Another approach was proposed in article [17], where a comfort index was introduced to allow its assessment separately for each vibration frequency band. The value of this index is expressed by dividing the effective acceleration value by the corresponding comfort limit defined in the earlier edition of the ISO 2631-1 standard. The proposed method, therefore, makes it possible to compare the level of comfort resulting from the impact of vibrations of specific frequencies and directions.

As seen above, ride comfort regarding the impact of mechanical vibrations on road and rail transport vehicle passengers has been the subject of many analyses. In the current situation, when there is a clear tendency to increase the speed of trains and introduce newer rolling stock and electric cars, the issue of comfort remains an essential subject of on-going investigations. In addition, due to the multitude of stimuli affecting passengers during a journey, it is advisable to develop and apply assessment methods that take into account more than just one type of comfort.

Previously published reviews and research not related to vibration comfort are usually focused on one selected factor, often without determining the level of passenger satisfaction with the journey. Thermal comfort was considered, for example, in studies [18,19,20,21,22]. Other studies concern acoustic comfort [23,24,25], air quality [26], pressure [27], and visual comfort [28].

The current state of the literature presents few solutions that would consider a greater number of factors or introduce a comprehensive comfort index. One of the attempts to introduce a new objective assessment method was an index determined based on vibration and noise measurements [29]. However, the methodology proposed in that article does not take into account the feelings associated with the thermal environment or lighting conditions. Study [30] presents a more complex approach, which presents five groups of different factors: vehicle interior, microclimate, vehicle operation, congestion level, and visual comfort. Other components were assigned to each group. However, the publication is more of an overview since no clear assessment criteria or methods for determining the comfort index have been specified. An attempt to create a synthetic comfort indicator was also made in study [31], which integrates data on humidity and air temperature, vibrations, air quality (CO_2_ concentration), noise, and illuminance. Comfort levels have been established for each factor based on public health norms and standards. The final overall indicator is determined using a geometric mean of the components determined separately for each factor. The method has been proposed as a complementary tool for the management and planning of public transport. Classic transport management models mainly take into account the time and cost of travel. The authors point out that this type of comfort indicator could support decision-making by relevant institutions to optimally allocate funds for rolling stock maintenance or modernization and repair. To validate the method, experimental studies were carried out, and for comparison, surveys were conducted, both in new and older vehicles.

Each type of ride comfort can be influenced by several minor factors. In the case of vibrational comfort, these include acceleration, frequency, direction, and location of vibration, as well as exposure time. Thermal comfort consists of factors related to the vehicle, including especially heating, ventilation, and air conditioning (HVAC) systems and crowding. It is also important where the vehicle operates, and whether solar radiation is present. The sensations associated with this type of comfort will also be influenced by the season of the year and the ambient temperature. In addition, factors directly related to humans are important, such as clothing or metabolic rate. The feeling of visual comfort will be influenced by the light temperature, encompassing the maintenance of the luminaries as well as their age and wear. The presence of natural light is also important.

According to the above literature review, passenger comfort is most often assessed according to one selected factor. A significant part of the research to date is focused on the impact of mechanical vibrations on passenger satisfaction with the journey. Due to the growing importance of this issue, it is advisable to expand the research by considering other relevant factors.

## 3. Methods for Ride Comfort Assessment

### 3.1. Mechanical Vibration

Vibration acceleration and frequency are the main parameters characterizing vibrations, used to assess their impact on ride comfort. The direction and time of impact, as well as the place of application, are also important. The influence of vibrations on comfort sensations can be approximated using synthetic indicators, determined based on measured or simulated vibration acceleration waveforms. These indicators are expressed in terms of effective (RMS) values of the averaged over the frequency domain by weight functions. These functions are linked to the body’s different responses to vibrations of various frequencies so that components with frequencies considered less harmful are adequately suppressed. The value obtained in this way is usually referred to as a multi-point descriptive scale. One of the first indicators of this type was the WZ index proposed by Sperling [32]. Standards widely used for the assessment of vibration comfort are UIC 513 [33], BS 6841:1987 [34], ISO 2631-1:1997 [6], and EN 12299:2009 [12]. These standards may differ in rating scales or weight functions, but most of them are used for vibration with frequencies of the one-third octave bands in the range from 0.8 Hz to 80 Hz or up to 100 Hz. The standards take into account different human positions (standing, sitting) and the planes of contact of the human body with vibrating elements. This may be, for example, floor-feet or seat-buttocks interfaces.

**Figure 1:**
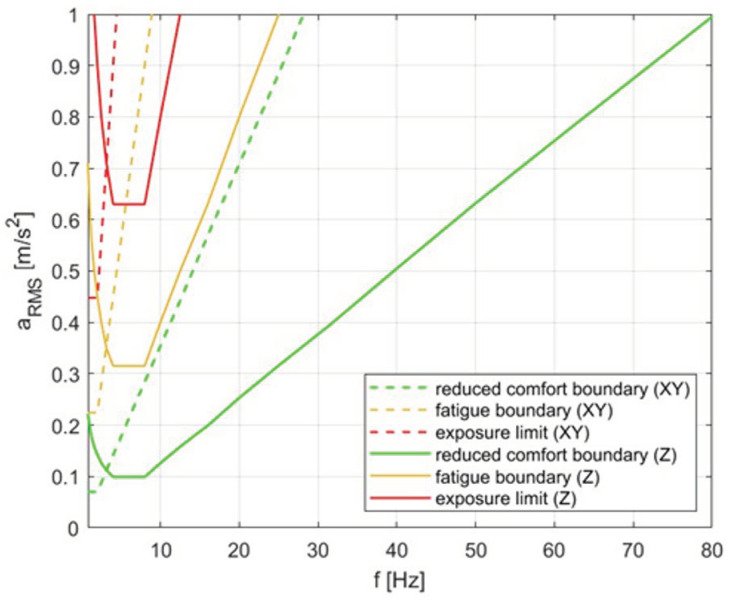
Reduced comfort boundary, fatigue-decreased proficiency boundary, and exposure limit for horizontal vibration (X, Y) with a dashed line and for vertical vibration (Z) with a solid line according to ISO 2631:1-1987 [6].

The ISO 2631-1 document [6] in the 1985 edition introduced recommendations for spectral analysis of the RMS values of vibration accelerations in the one-third octave bands. In this method, the obtained test results are compared with the reduced comfort boundaries, fatigue-decreased proficiency boundaries, and exposure limits. The limit values given in the standard depend on the center frequencies of one-third octave bands and have been defined separately for horizontal and vertical vibrations. The range of these frequencies is 0.8–80 Hz. The corresponding boundaries are shown in Figure 1.

A later edition of the standard from 1997 introduced a synthetic comfort index. For a seated person, two weight functions have been defined: *W_d_* is used for the values of effective accelerations of longitudinal and transverse vibrations, and *W_k_* is used for vertical vibrations. The RMS values of vibration accelerations in the one-third octave bands are averaged separately for each direction of the vibration according to Equation (1):

(1) $$a_{{\eta ,}_{RMS-w}}=\sqrt{\sum_{i=0}^{n}{\left[W_{d}\left(f_{i}\right)\cdot a_{{\eta ,}_{RMS}}\left(f_{i}\right)\right]}^{2}}\left(\eta =X,Y\right)$$

where:

- $W_{d}\left(f_{i}\right)$—weight function depending on the center frequency of the one-third octave bands ranging from 0.8 Hz to 80 Hz (*n*—number of the bands),
- $a_{{\eta ,}_{RMS}}\left(f_{i}\right)$—*RMS* values of vibration acceleration in the consecutive one-third octave bands in direction *X* or *Y*, respectively.
- $a_{{\eta ,}_{RMS-w}}$—averaged *RMS* value of vibration acceleration for the transverse direction of vibration propagation.

In the case of vertical vibrations, an analogous relationship is used, with the change of the weighting function to *W_k_*. The final value of the comfort index is given by the total weighted effective value of acceleration calculated from Formula (2) and is expressed in m/s^2^.

(2) $$a_{RMS-w}=\sqrt{a_{x,RMS-w}^{2}+a_{y,RMS-w}^{2}+a_{z,RMS-w}^{2}}$$

The ISO 2631-1 standard [4] defines six levels of ride comfort, from “comfortable” (*a_RMS-w_* ≤ 0.315 m/s^2^), through “slightly uncomfortable” (0.315 m/s^2^ < *a_RMS-w_* < 0.630 m/s^2^), “quite uncomfortable” (0.500 m/s^2^ < *a_RMS-w_* < 1.000 m/s^2^), “uncomfortable” (0.800 m/s^2^ < *a_RMS-w_* < 1.600 m/s^2^), “very uncomfortable” (1.250 m/s^2^ < *a_RMS-w_* < 2.500 m/s^2^), to “extremely uncomfortable” (*a_RMS-w_* > 2.000 m/s^2^).

### 3.2. Air Temperature and Relative Humidity

Thermal comfort can be defined as a state of mind that expresses satisfaction with the thermal environment. The factors influencing this type of comfort can be assigned to two groups. The first of these are physical parameters describing the environment in which people reside, such as air temperature, relative humidity, and ventilation flow rate, as well as the temperature of walls and objects in the environment. To the second group, we assign factors directly related to humans, i.e., metabolic rate, human skin temperature, and thermal insulation of clothing. It is, therefore, impossible to determine parameters of the thermal environment that would be satisfactory for everyone, which is why the standards for its assessment usually contain ranges of values acceptable for the sake of comfort.

The requirements for the thermal environment in rail vehicles are formulated in the European standard EN 14750-1:2006 [35]. This document recommends temperature ranges and corresponding air velocity ranges. On the other hand, in the UIC 553:2004 leaflet [36], these ranges are defined separately for the summer and winter seasons. The above standards refer only to the above-mentioned environmental parameters and do not consider factors directly related to humans.

One of the first models to describe thermal comfort in a more detailed manner was the PMV (Predicted Mean Vote) indicator that was introduced in 1970 by P. O. Fanger [37]. This model was based on human thermal equilibrium. The indicator combines environmental and individual variables, thus representing the average predicted comfort rating on a seven-point thermal experience scale. The PDD (Predicted Percentage of Dissatisfied) is related to the above index, which refers to the predicted percentage of people dissatisfied with the thermal environment. Based on Fanger’s model, commonly used standards for assessing human indoor thermal comfort are ISO 7730:2005 [38] and ASHRAE 55 [39]. It should be noted that the PMV indicator is a function of many variables, and its determination requires complex measurements using a set of sensors.

**Figure 2:**
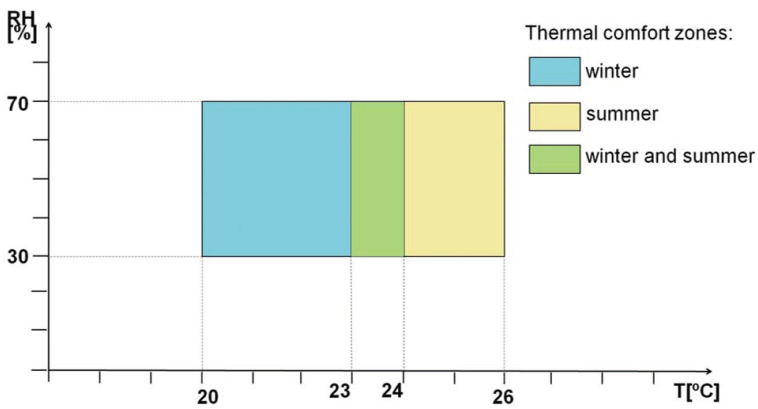
Thermal comfort zones according to ISO 7730:2005 [38].

Among the numerous factors affecting thermal comfort, the most important should be indicated as air temperature and relative air humidity [31]. Too high a level of humidity can intensify negative feelings related to temperature. The ISO 7730:2005 standard [38] proposes a simplified approach that can be applied to less advanced analyses and that takes into account these main controllable factors. The document defines the so-called comfort zones separately for the summer and winter seasons. The ranges of recommended air temperatures and relative humidity in accordance with the standard are shown in Figure 2. Although this standard applies to buildings, it can also be used to analyze thermal comfort in vehicles.

### 3.3. Lighting

Visual comfort refers to the light environment, which consists of the level of natural and artificial lighting. The interior of the vehicle and the light source’s color are also essential and should be as neutral as possible to maintain optimal visual effects.

The factors for this type of comfort can also include the parameter that represents the light source, for example, the color rendering index CRI (color rendering index). This quantity is a measure of the ability of a light source to reproduce the colors of individual objects (e.g., seats, handrails) [40]. In addition, one can distinguish the phenomenon of uncontrolled and excessive brightness appearing in the field of view, i.e., glare phenomena. The occurrence of glare generally does not impair visual conditions but may contribute to visual discomfort for the passenger [41].

Although all the factors mentioned above affect visual comfort, the level of illuminance is the most important and necessary to perform various activities. This quantity is expressed by the ratio of the luminous flux incident on the plane to the surface area of this plane, and its unit is lux (lx). The illumination level mainly determines the comfort of vision, encompassing the ability to recognize details and the convenience of performing visual tasks such as reading.

When assessing lighting in rail vehicles, standards such as the UIC 651:2002 [42], EN 13272-1:2019 [43], and EN 13272-2:2019 [44] are used. The UIC 651:2002 leaflet refers only to the illuminance of the driver’s desk, which shall not be less than 60 lux. European standards, in addition to setting minimum requirements for the uniformity of illuminance, glare, light temperature, and color rendering index, specify the values of minimum illumination in long-distance railway vehicles and in urban rail vehicles, respectively. In both documents, the permissible minimum illumination levels are defined with a distinction between different zones (Table 1).

## 4. Experimental Research

### 4.1. Description of the Test

For the experimental research, Alstom Metropolis 98B electric multiple units were used. This vehicle is a part of the Warsaw Metro rolling stock operating on the first metro line. The operation of the first trains began in 2000, and additional ones were introduced to the rolling stock gradually until 2005. Each train consists of two trailer and four motor cars which can accommodate 229 and 249 passengers, respectively.

The research experiment was based on measurements of parameters characterizing the following factors: acceleration of vibrations acting in three directions, air temperature, relative air humidity, and illuminance.

**Figure 3:**
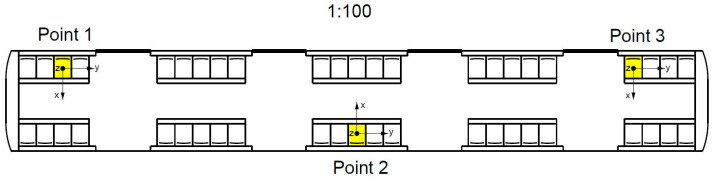
Spatial distribution of seats in the Metropolis 98B metro motor car with measuring points.

The tests were carried out during the journey from the Marymont station to the Racławicka station. Three measuring points were chosen. These were located at the front, rear, and middle of the motor car. The location of the points is marked in Figure 3.

The vibration accelerations of the metro car were measured on the seats in the transverse (X, Y) and vertical (Z) directions oriented according to the ISO 2631:1 coordinate system [6]. The sensor was mounted on the seats with strong tape.

The entire system of the Warsaw metro runs through underground tunnels, so the influence of natural light was not taken into account for the tested metro car. This parameter was measured 800 mm above the floor level, as recommended by the EN 13272-2:2019 standard [44] for urban rail vehicles (Table 1). In the context of thermal comfort factors, it is important to mention that this research was conducted in the transitional period between the winter and spring seasons. All measurements were carried out under typical running conditions in metro cars carrying passengers.

### 4.2. Measuring Equipment

To conduct experimental research, a modern mobile measuring instrument S3-D40 was used. The device was manufactured by enDAQ (Woburn, United States). It is equipped with a capacitive accelerometer, registering vibration accelerations in three directions. The measurement range is ±40 g, sampling rate 4 kHz, bandwidth from 0 to 300 Hz, and resolution 0.00008 g. The measuring device had the valid manufacturer’s calibration at the time of making the measurements.

In addition to the accelerometer, the device is equipped with additional sensors enabling simultaneous measurements of other quantities characterizing environmental factors. The same device was used to measure air temperature, relative air humidity, and illuminance. The additional sensors of the measuring instrument S3-D40 delivered by enDAQ are:

- temperature sensor FXPQ3115 by NXP, with measurement range from −40 to 80 °C, resolution 0.01 °C, and sampling rate up to 10 Hz,
- air humidity sensor HTU21D by MEAS, with measurement range from 0 to 100%, resolution 0.04%, and sampling rate up to 10 Hz,
- illuminance sensor SI1133 by Silicon Labs, with resolution less than 100 mlx, and sampling rate up to 4 Hz.

The measuring device was securely mounted on the passenger seat with the double-sided tape (3M 950) supplied for this purpose by the device manufacturer. Such a mounting method is adequate due to the compact size of the device, with the length of 76.2 mm, the width of 29.8 mm, the height of 15 mm, and the low mass of 40 g. The manufacturer’s enDAQ Lab software was used to preview and export the measurement data. The calculation of the weighted effective values of the vibration accelerations was carried out using a script in the MATLAB version: 9.13.0 (R2022b) [45].

## 5. Analysis of the Test Results

The criteria for assessing ride comfort were selected based on the recommendations provided by the relevant standards. In the case of mechanical vibrations, the spectral method compliant with ISO 2631:1 [6] was used to determine at which frequencies of the one-third octave bands RMS values of vibration accelerations exceed the limits permissible for passenger comfort for every direction of vibration impact. In addition, the weighted method was used in line with the recommendations of the subsequent issue of the same standard. On this basis, synthetic indicators were determined, which were related to the comfort scale. The method of determining indicators and information about the scale is discussed in Section 3.1.

In the case of thermal comfort assessment, a simplified method was used, which does not require a complicated set of measuring equipment. The assessment criterion is the comfort zone defined in ISO 7730:2005 [38] for the winter season. The average values of air temperature and relative humidity measured in the three vehicle zones are referred to the permissible ranges of these values, as in Figure 2.

The assessment of visual comfort was based on the most important factor influencing this type of comfort: illuminance. Following the recommendations of the EN 13272-2:2019 standard [44], a minimum level suitable for seating areas is assumed.

**Figure 4:**
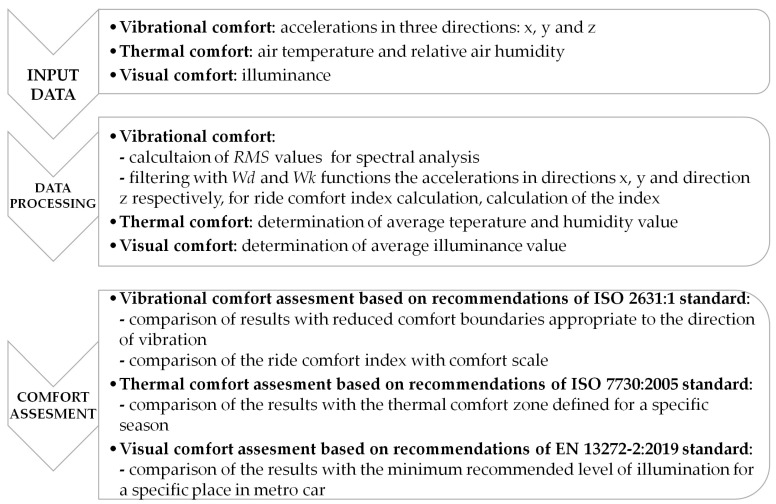
The process of ride comfort assessment.

The obtained results were then compared with results from the Montreal metro system published in study [31]. The graphical scheme of the ride comfort assessment in the Warsaw metro used in this study is shown in Figure 4.

### 5.1. Vibration Comfort Test Results

**Figure 5:**
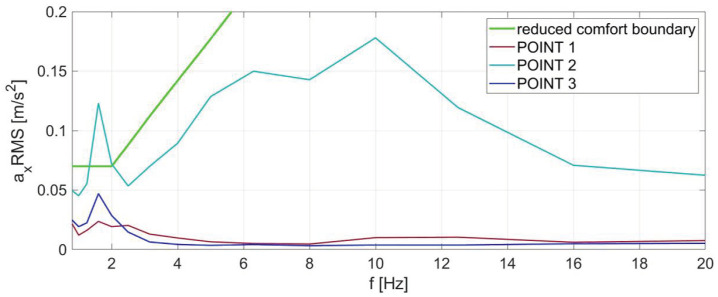
RMS values of vibration accelerations in the one-third octave bands for the metro car for direction X at three measuring points, together with the ISO 2631:1 reduced comfort boundary.

Figure 5 shows the results of the RMS vibration acceleration values in the one-third octave bands for the metro car under test at three measuring points. The presented values correspond to vibrations affecting the passenger in the direction X. At frequencies higher than 20 Hz, no exceedances of the reduced comfort boundary were found. Therefore, for better visibility of the results, the graphs have been limited to the 0.8 Hz to 20 Hz range.

The results for transverse vibrations show a slight excess of the reduced comfort boundary in the metro car for the second measuring point located in the middle of the vehicle. This may be due to frequent starting and stopping of the vehicle at metro stations. The remaining results do not indicate a decrease in the level of passenger ride comfort according to the ISO 2631:1 recommendations.

**Figure 6:**
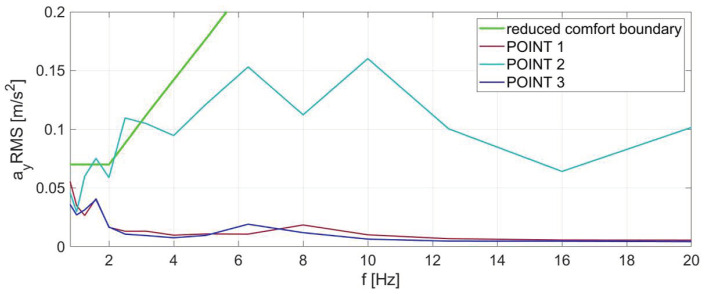
RMS values of vibration accelerations in the one-third octave bands of the metro car for the Y direction at three measuring points, together with the ISO 2631:1 reduced comfort boundary.

The results for direction Y are shown in Figure 6. Similarly, as for the X direction, the range of presented results was limited to vibrations with frequencies of the middle one-third octave bands not exceeding 20 Hz. The RMS values of accelerations determined based on recordings in the same metro car show a slight exceeding of the comfort boundary at low frequencies. These exceedances occur only at the second measuring point (the middle part of the car), as it was in the case of transverse vibration.

**Figure 7:**
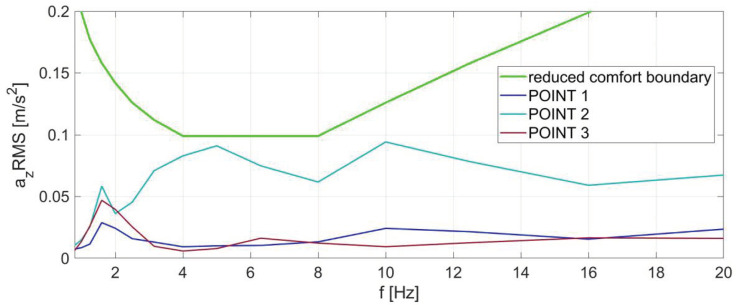
RMS values of vibration accelerations in the one-third octave bands of the metro car for the Z direction at three measuring points, together with the ISO 2631:1 reduced comfort boundary.

The results for direction Z are shown in Figure 7. In contrast to the results presented in the previous graphs, there is a significant difference in the RMS values of the vibration accelerations. The boundary defined in the ISO 2631:1 standard is not exceeded in any of the one-third octave bands at any of the measuring points. Such RMS values of vibration accelerations might be noticeable to passengers but would not cause discomfort. It should be noted that vertical vibrations are usually the cause of lower ride comfort, and therefore the presented results can be considered as very satisfactory.

The ride comfort indexes calculated using the ISO weighted method were based on the same values of effective vibration accelerations, as shown in Figure 5, Figure 6 and Figure 7. The results are presented in Table 2.

According to the presented results, the journey on the seats located at the front and rear of the vehicle should be described as “comfortable”. In the case of the second measuring point located in the middle of the car, the index indicates a “slightly uncomfortable” ride with longitudinal and vertical vibrations having the most significant contribution to reducing the comfort level, as shown by the results of the indicator components. Given the above, the overall vibration comfort in the tested metro car is mostly at an acceptable level.

In the assessment of vibrational comfort in the Montreal metro system [31], the researchers did not use the spectral method. Therefore, it is not possible to compare the results in terms of vibration frequency. However, they did use the recommendations of the ISO 2631:1 standard for the determination of the comfort index. The method was modified by adding a new seven-point scale with weights assigned to each point, in order to develop a new customized comfort index which has been marked with the symbol V_I_ (non-nominal) with no unit. The results of the ride comfort assessment were reported w according to the proposed scale, and therefore the direct comparison is hindered, but not completely impossible. Most of the results for the new metro cars show that the V_I_ index varies from 0.7 to 0.9, and for older cars from 0.5 to 0.7. This translates to the ISO standard comfort index *a_RMS-w_* values from 0.315 m/s^2^ to 0.750 m/s^2^, and from 0.750 m/s^2^ to 1.000 m/s^2^ respectively. According to the ISO standard, the results for the new cars fall into the categories of “slightly uncomfortable” or “quite uncomfortable”. Given the above range of possible *a_RMS-w_* index values, the comfort level cannot be clearly stated. The same situation applies to older subway cars, but the comfort levels vary from “quite uncomfortable” to “uncomfortable”. Comparing the two metro systems on the above basis, it can be concluded that Warsaw metro cars provide better vibration comfort.

### 5.2. Thermal Comfort

**Figure 8:**
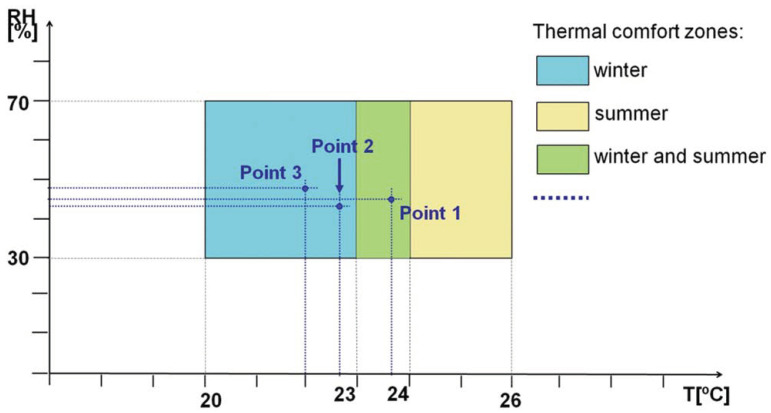
Thermal comfort zones according to ISO 7730:2005 with an average value of air temperature and relative humidity in the subway car.

Figure 8 shows the results of average air temperature and relative humidity at all measuring points, together with thermal comfort zones according to the ISO 7730:2005 standard [35].

All measurements were carried out in the winter season. Therefore, the results should be applied first to the area marked in blue and then to the area marked in green, which refers to good thermal comfort in both seasons, as distinguished in the ISO 7730:2005 standard. Table 3 summarizes the numerical results with the corresponding values for the vehicle’s surroundings. The parameters were recorded at a station located in an underground tunnel.

The results of experimental studies show that good thermal comfort conditions were provided. All measurements are within the comfort zone defined in the ISO 7730:2005 standard. The average value of relative humidity is optimal in every measuring point. Higher temperature was observed at the end of the car. The average value was in the zone of thermal comfort provided in the standard for both seasons. However, it cannot be said that taking a seat in this part of the metro car may decrease thermal comfort.

Comparing the results of the metro in Montreal and Warsaw in winter, it can be concluded that in the second city better conditions of thermal comfort were provided. Most of the results from Montreal showed temperatures higher than 25 °C and relative air humidity less than 20% [31]. These results do not fall into the ranges of the thermal comfort zone defined in the mentioned standard.

### 5.3. Visual Comfort

**Figure 9:**
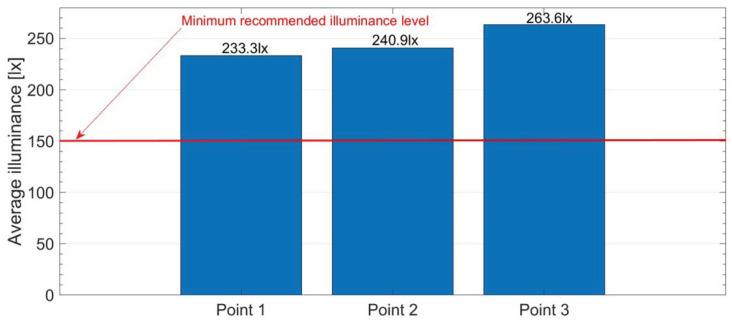
Illuminance results measured in the metro car at three measuring points together with the minimum recommended value of this parameter.

Figure 9 shows the results of illuminance measurements in metro cars, together with the indication of the minimum recommended value of this parameter in the EN 13272-2:2019 standard [44].

According to the data in Figure 9, the minimum illuminance level requirements were met in the tested metro car. Satisfactory lighting conditions and, therefore also, a good level of visual comfort were provided at each of the three measuring points. This situation is undoubtedly due to the arrangement of luminaires placed on the metro car ceiling. The lighting of the passenger compartment in metro cars consists of two symmetrically distributed rows of these devices, which as confirmed by experiment is enough to ensure a good lighting environment.

Warsaw metro cars rank between old and new Montreal metro cars. According to results published in article [31], for older metro cars the illuminance level is less than 200 lx. The authors assumed that the best lighting environment is provided by illuminance above 300 lx. This assumption was met for new vehicles for which no specific numerical value was provided, but it was pointed out that it exceeded the most comfortable threshold. It should be noted that these requirements differ from those presented in the study. However, by comparing tested Warsaw metro cars (which can be considered older) with those in Montreal, it can be stated that passengers are provided with a better level of visual comfort.

## 6. Summary and Conclusions

This article presents methods for assessing ride comfort and the results of experimental tests carried out in a selected metro car. Due to the growing importance of this issue, an attempt was made to broaden the assessment of factors affecting the feeling of passenger comfort, taking into account not only the influence of dynamic interactions but also the temperature and relative air humidity and illuminance. These factors were assessed based on the recommendations of commonly used standards. The most important findings of this research are as follows:

- Based on the ride comfort indicators determined by the weighted method, it can be stated that the tested metro car provides “comfortable” conditions, with the lowest synthetic comfort index of 0.111 m/s^2^ for the first measuring point located at the end of the wagon, and a slightly higher value of 0.113 m/s^2^ at the front of the wagon. The indicators for the measuring points located at the front and rear of the vehicle reach the lowest values. Slightly worse conditions due to the impact of vibrations are provided by the central part of the vehicle with a rating of “slightly uncomfortable”. The results of the weighted components of effective vibration acceleration indicate the greatest impact of vertical vibration on lower ride comfort level.
- Similar conclusions about vibration comfort can be drawn from a spectral analysis of vibration accelerations. The RMS values of vibration accelerations in the one-third octave bands calculated based on the measured accelerations in the subway car indicate the possibility of lowering the comfort level only in the central part of the vehicle (second measuring point). Exceedances of the reduced comfort boundary occurred only in the case of lateral and longitudinal vibration in the middle frequencies of the one-third octave bands of 1.6 Hz and 2.5 Hz. In these bands, the boundary is 0.070 m/s^2^ and 0.088 m/s^2^, respectively. The obtained results are 0.075 m/s^2^ and 0.110 m/s^2^. The results for transverse vibration showed overrun in the band with the middle frequency of 1.6 Hz with the RMS value of 0.12 m/s^2^ and the same boundary as for longitudinal vibration. The vertical vibration did not contribute to passenger discomfort. Given the above, the overall vibration comfort in the tested metro car is at a good level. Particular attention should be paid to low frequency vibration.
- At every measuring point, passengers were provided with good thermal comfort. The average air temperatures in the wagon ranged from 22.0 °C to 23.6 °C, with the highest temperature at the front part of the wagon (first measuring point). In the case of average values of relative air humidity, larger differences in results were observed. The lowest value of 42.2% was recorded in the center of the wagon, i.e., at the second measuring point. The highest average air humidity was measured at the end of the wagon with the value of 47.1%. The thermal comfort zone defined for winter defines the recommended air temperatures in the range of 20 °C to 24 °C and relative air humidity in the range of 30% to 70%, regardless of the season. Although the average values of air temperature and relative humidity vary depending on the vehicle’s passenger seat, the results fall within the thermal comfort zone. It should be noted that thermal comfort also consists of other factors related to the environment as well as to the individual characteristics of people and their clothing or activity. A more detailed assessment of this type of comfort is recommended especially in areas with demanding climatic conditions.
- Illuminance measurements indicate satisfactory visual comfort conditions in the subway car. The average results ranged from 233.3 lx to 263.3 lx, with the highest value recorded at the third measuring point. The minimum requirements of the EN 13272-2:2019 standard [44], which for seating and standing areas should be at least 150 lx, were met in the tested metro car. The lighting environment in the tested metro car shall be sufficient to perform simple visual tasks such as reading. It should be noted, however, that the recommended value of the illuminance level in the standard should only be the initial starting point for comparing lighting conditions in different rail vehicles because the standard does not directly specify the threshold for the sake of comfort.

Experimental research shows that while the overall level of ride comfort of the tested metro car can be described as satisfactory, it should still be pointed out that vibrations occurring during a journey can contribute to its reduction. Furthermore, for deeper analysis of thermal comfort it is advisable to repeat the experiment at a different season. In the case of visual passenger feelings, illuminance measurements are important in the context of assessing the wear of luminaires. In fact, in the case of completely non-functioning luminaires, detecting a problem by the vehicle staff is simple. However, gradual wear and tightness along with the possibility of tarnishing and dustiness can affect the deterioration of the light environment, especially in older vehicles.

The assessment of many factors simultaneously can support monitoring the quality of services for transport providers. The presented research shows relatively uncomplicated methods for assessing vibration, thermal, and visual passenger comfort. The advantage of these methods is that they can be used for initial evaluation of comfort even by untrained workers. Identifying the factors that contribute most to the reduction in comfort levels points to areas for improvement or further investigation. Using ride comfort assessment can also be useful for track monitoring. The samples of vibration signals from the same train running at different speeds and on different lines and tracks together with geolocation data can bring specific information about the technical condition of the track. The significance of ride comfort lies also in increasing the expectations of passengers, but what is more important is how it can be an incentive factor to change the habits of society. Following the strategies of the European Union, there is an urgent need for reducing the use of personal modes of transportation and for shifting the greater part of urban mobility to public transport. More frequent use of the metro system can contribute to lower congestion and emissions of air pollutants, which will cause a reduction in the external costs of transport and better public health. Therefore, research on a wider group of ride comfort factors remains a current topic.

Although the presented methods have been applied to metro cars, they can be applied to other modes of public transport, such as trams and long-distance trains or buses. However, the method has some limitations. It is advisable to consider the environment in which those vehicles operate and also the differences in the interiors of different transport modes. For example, for vehicles operating in open spaces visual comfort might not be as important in daylight. In addition, vehicles can vary in the arrangement of seats. The seats can be arranged according or opposite to the direction of movement. This requires adjusting the vibration acceleration measurements to the coordinate system of the given standard. Another limitation concerns thermal comfort. The entire Warsaw metro system operates in underground tunnels so there is no effect of solar radiation coming through the windows. For other modes of transport, this may be an important factor affecting passengers’ feelings.

Other directions of this research include repeating the experiment in different time of the year with all types of Warsaw metro cars. This research took place only in the first and oldest metro line. It would be useful to compare results from both of the lines. To more accurately assess the comfort level, it is advisable to assess other stimuli such as noise, air quality, or pressure. However, passengers may also be affected by factors which are difficult to measure and are strongly dependent on subjective perception. Those factors may be cleanliness or passenger crowding. This article took into consideration only chosen factors that describe the internal environment of the metro car. Future research plans involve the development and conduct of surveys among large research group distinguishing between age group, gender, and the purpose and frequency of metro journeys. The surveys will be aimed at ranking measurable and non-measurable factors to be able to assign each of them the appropriate importance.

## Figures and Tables

**Table 1:** Recommendations of EN 13272:2019 [43,44] regarding illuminance in rail vehicles.

Standard	Location in the Unit	Height above Floor Level	Minimum Illuminance *E*
EN13272-1:2019 Heavy and high-speed units	Seating areas where no additional reading lights are provided	Passenger eye level	≥150 lx
	Seating areas with reading lights which are switched off		≥100 lx
	Side corridors and aisles	At floor level	≥50 lx
		At 0.8 m above floor level	≥75 lx
	Standing areas, multifunctional areas, open gangways	At 0.8 m above floor level	≥75 lx
EN13272-2:2019 Urban rail vehicles	Seating areas	At 0.8 m above floor level	≥150 lx
	Standing areas	At 0.8 m above floor level	≥150 lx
	Open gangways	At floor level	≥50 lx

**Table 2:** Ride comfort indexes in the metro car for three measuring points.

Comfort Index [m/s^2^]	Measuring Points		
	Point 1	Point 2	Point 3
*a_x,RMS-w_*	0.045	0.202	0.068
*a_y,RMS-w_*	0.085	0.183	0.071
*a_z,RMS-w_*	0.055	0.206	0.057
*a_RMS-w_*	**0.111**	**0.342**	**0.113**
Comfort level	comfortable	Slightly uncomfortable	comfortable

**Table 3:** Results of average temperature and relative air humidity values in the metro car.

Measuring Point	Average Air Temperature T [°C]	Average Relative Humidity RH [%]	Environment	
			T [°C]	RH [%]
Point 1	23.6	43.8	22.3	40.7
Point 2	22.6	42.2		
Point 3	22.0	47.1		

## Data Availability

Not applicable.

## References

[1] Passenger Transport Statistical Portal of the Office of Rail Transport (In Polish). https://dane.utk.gov.pl/sts/przewozy-pasazerskie/dane-eksploatacyjne/19890,Przewozy-pasazerskie.html.

[2] Jacyna-Gołda I., Gołębiowski P., Izdebski M., Kłodawski M., Jachimowski R., Szczepański E. (2017). The evaluation of the sustainable transport system development with the scenario analyses procedure. J. Vibroeng..

[3] (2002). Transportation—Logistics and Services—Public Passenger Transport—Service Quality Definition, Targeting and Measurement.

[4] Kardas-Cinal E. (2020). Statistical Method for Investigating Transient Enhancements of Dynamical Responses due to Random Disturbances: Application to Railway Vehicle Motion. ASME. J. Vib. Acoust..

[5] Zhou Y., Chen S. (2016). Vehicle ride comfort analysis with whole-body vibration on long-span bridges subjected to crosswind. J. Wind Eng. Ind. Aerodyn..

[6] (1997). Mechanical Vibration and Shock-Evaluation of Human Exposure to Whole-Body Vibration, Part 1: General Requirements.

[7] Wawryszczuk R., Kardas-Cinal E. (2021). Analysis of ride comfort in selected types of rail vehicles. J. KONBiN.

[8] Sekulić D., Dedović V., Rusov S., Obradović A., Šalinić S. (2016). Definition and determination of the bus oscillatory comfort zones. Int. J. Ind. Ergon..

[9] Kırbaş U., Karaşahin M. (2021). Discomfort limits provided by railroad crossings to passenger cars. Int. J. Pavement Eng..

[10] Wu J., Qiu Y. (2021). Analysis of ride comfort of the high-speed train based on a train-seat-human model in the vertical direction. Veh. Syst. Dyn..

[11] Bakinowski Ł., Firlik B. (2022). Influence of the type of place a tram passenger occupies on the ride comfort. Arch. Transp..

[12] (2009). Railway Applications—Ride Comfort for Passengers—Measurement and Evaluation.

[13] Ján D., Miroslav B., Juraj G., Leitner B., Melnik R., Semenov S., Mikhailov E., Kostrzewski M. (2021). Evaluation of Ride Comfort in a Railway Passenger Car Depending on a Change of Suspension Parameters. Sensors.

[14] Park J., Lee J., Ahn S., Jeong W. (2017). Reduced ride comfort caused by beating idle vibrations in passenger vehicles. Int. J. Ind. Ergon..

[15] Huang Y.U., Li D. (2019). Subjective discomfort model of the micro commercial vehicle vibration over different road conditions. Appl. Acoust..

[16] Barone V., Mongelli D.W.E., Tassitani A. (2016). Vibrational comfort on board the vehicle: Influence of speed bumps and comparison between different categories of vehicle. Adv. Acoust. Vib..

[17] Kardas-Cinal E., Kardas-Cinal E., Korzeb J. (2017). Analysis of the ride comfort of a rail vehicle passenger—A proposal for an indicator depending on vibration frequency. Selected Issues of Testing Dynamic Interactions in Rail Transport.

[18] Moon J.H., Lee J.W., Jeong C.H., Lee S.H. (2016). Thermal comfort analysis in a passenger compartment considering the solar radiation effect. Int. J. Therm. Sci..

[19] Yakovenko Y., Voichyshyn Y., Horbay O. (2022). Analysis of thermal comfort models of users of public urban and intercity transport. Ukr. J. Mech. Eng. Mater. Sci..

[20] Danca P., Vartires A., Dogeanu A. (2016). An overview of current methods for thermal comfort assessment in vehicle cabin. Energy Procedia.

[21] Hintea D., Kemp J., Brusey J., Gaura E., Beloe N. Applicability of thermal comfort models to car cabin environments. Proceedings of the 2014 11th International Conference on Informatics in Control, Automation and Robotics (ICINCO).

[22] Pala U., Oz H.R. (2015). An investigation of thermal comfort inside a bus during heating period within a climatic chamber. Appl. Ergon..

[23] Khelf M., Boukebbab S. (2018). The effect of noise on the comfort of passengers inside the tramway and its impact on traffic congestion in the urban area. J. Vibroeng..

[24] Patania F., Gagliano A., Nocera F., Galesi A. (2013). Analysis of acoustic climate on board public transport. Environ. Health Risk VII.

[25] Park B., Jeon J.Y., Choi S., Park J. (2015). Short-term noise annoyance assessment in passenger compartments of high-speed trains under sudden variation. Appl. Acoust..

[26] Li T.T., Bai Y.H., Liu Z.R., Li J.L. (2007). In-train air quality assessment of the railway transit system in Beijing: A note. Transp. Res. Part D Transp. Environ..

[27] Schwanitz S., Wittkowski M., Rolny V., Basner M. (2013). Pressure variations on a train–Where is the threshold to railway passenger discomfort?. Appl. Ergon..

[28] Xu J., Xiang Z.R., Zhi J.Y., Chen Y.D., Xu X.F. (2022). Assessment of visual comfort in the lighting environments of subway cabins in China. Int. J. Rail Transp..

[29] Amador-Jimenez L., Christopher A. A comfort index for public transportation: Case study of Montreal. Proceedings of the 2016 IEEE International Conference on Intelligent Transportation Engineering (ICITE).

[30] Xu J. (2021). Build a Comprehensive Ride Comfort Index System for Subway Trains. Converter.

[31] Mohammadi A., Amador-Jimenez L., Nasiri F. (2020). A multi-criteria assessment of the passengers’ level of comfort in urban railway rolling stock. Sustain. Cities Soc..

[32] Sperling E., Betzhold C. (1956). Contribution to evaluation of comfortable running of railway vehicles. Bull. Int. Railw. Congr. Assoc..

[33] (1994). Guidelines for Evaluating Passenger Comfort in Relation to Vibration in Railway Vehicle.

[34] (1999). Guide to measurement and evaluation of human exposure to whole body mechanical vibration and repeated shock.

[35] (2006). Railway Applications—Air Conditioning for Urban and Suburban Rolling Stock—Comfort Parameters.

[36] (2004). Heating, Ventilation and Air-Conditioning in Coach.

[37] Fanger P.O. (1970). Thermal Comfort: Analysis and Applications in Environmental Engineering.

[38] (2005). Ergonomics of the Thermal Environment—Analytical Determination and Interpretation of Thermal Comfort Using Calculation of the PMV and PPD Indices and Local Thermal Comfort Criteria.

[39] (2020). Thermal Environmental Conditions for Human Occupancy.

[40] Boyce P.R. (2014). Human Factors in Lighting.

[41] Quek G., Wienold J., Khanie M.S., Erell E., Kaftan E., Tzempelikos A., Andersen M. (2021). Comparing performance of discomfort glare metrics in high and low adaptation levels. Build. Environ..

[42] (2002). Layout of Driver’s Cabs in Locomotives, Railcars, Multiple Unit Trains and Driving Trailers.

[43] (2019). Railway Applications—Electrical Lighting for Rolling Stock in Public Transport Systems—Part 1: Heavy Rail.

[44] (2019). Railway Applications—Electrical Lighting for Rolling Stock in Public Transport Systems—Part 2: Urban Rail.

[45] (2022). Version 9.13.0 (R2022b).

